# Case of Premature Labor Complicated with Prolapsus of the Uterus

**Published:** 1850-08

**Authors:** 


*Case of premature labor complicated with prolapsus of the uterus.* By SAMUEL TYLER, M. D., of Fredericks, Md.

To S. S. PURPLE, M. D., Editor of the N. Y. Journal of Medicine.

*Dear Sir:*—The following case may not be without some interest to your readers, as it fully establishes the fact of the power of the muscular fibres of the uterus, and its ability to expel the fœtus or other contents, independently of assistance from any other source whatever. I am aware, of course, that at present there are few, if any, who deny its muscularity, if I be allowed the term, but it has not been many years since a distinguished teacher of obstetrics, in this State,* denied entirely the fact, and maintained that labor was effected by the agency of the muscular parietes of the abdomen.

On the 19th of March, 1846, at 2 o'clock, P. M., I was called to Mrs. M. I found with her a female midwife, who stated that the patient had had slight labor pains for several hours, and that there was a mass of fleshy substance between her thighs, which had "*come down*" early in the morning, when the patient was engaged in some work about the house. Upon examination, by the touch and by ocular inspection, I found, to my surprise, that it was the uterus entirely without the vagina, and containing a five months' fœtus; I could distinctly handle the lateral ligaments. To return it was impossible—the pains had somewhat subsided under the influence of opium, which had been administered by the midwife. Her pulse being full and active, I took sixteen ounces of blood from her arm, and administered twenty grains of powdered secale cornutum; in about twenty minutes the longitudinal fibres commenced contracting firmly, producing dilatation of the "os uteri;" they would cease, and the circular fibres would commence, producing an appearance of hour-glass contraction; the contraction of each lasting about a minute, by the watch. After the second contraction of the circular fibres, the womb was perfectly quiescent for about five minutes; when, as it were, by a concerted action of both sets of fibres, the fœtus, a male, was expelled and received in my hand, the secundines following in about ten minutes. I administered an anodyne, and requested that she be kept perfectly still until ten o'clock, P. M., when I visited her again, and was enabled to return the womb some distance up the vagina; administered another anodyne, and saw her at nine o'clock, A. M., the next day; she had rested well, and I found the womb nearly "in situ;" she kept in a pretty comfortable condition for four days, when puerperal fever ensued, with which she was ill about three weeks, but recovered perfectly, and has since been delivered of a fine child. I should have remarked that she had had four children previous, and is a woman of very delicate frame. I made the above notes of this case at the time, but my professional engagements have prevented, or rather caused me to neglect its publication until now.

### Remarks by the Editor.

The foregoing is the history of an exceedingly interesting case. Our home periodical literature, so far as our reading extends, records the particulars of two similar cases. In the first volume of the Medical Repository, for 1797, Dr. Thomas Archer, of Hartford-Town, Md., records the particulars of "A singular case of Difficult Parturition successfully treated." The following is a condensed account of the case. The patient, a Mrs. E., aged 30, had been in labor four days with her first child. Her pains had been considerably forcing, but the intervals between them long, the waters had been gradually discharging for two days. The os uteri was not dilated to more than the size of a cent, and it formed a thick, rigid cartilaginous ring, not yielding or becoming softened by the pain. Her constitution was robust and strong, and her pulse was marked by tension or convulsive action. She was bled to eighteen ounces, and gentle laxatives, with emolient clysters, were ordered; oleaginous injections into the

* Richard W. Hall, of the University of Maryland.

vagina, and the vapor of hot water were advised, as means for relaxing the os-tincæ. She took a dose of opium and stramonium, to procure rest and remove unprofitable pains. She was then left in the charge of a midwife until the following day in the evening.

At this time there was no perceptible alteration in the dilatation of the os uteri. The child's head could now with difficulty be felt through the os uteri, and there was no increase in the dilatation. A few pains protruded the uterus with its contents, without the os externum! The child, from certain evidences, was believed to be dead, and great fears were entertained for the patient's safety. Death appeared as the closing scene of every plan I proposed; in fact, her situation required such immediate assistance that there was scarcely a moment for deliberate thought. By means of a candle held by the midwife, without the knowledge of any other of the attendants, with a spear pointed lancet I made three incisions in the neck of the womb, each about two inches in length; viz., one leading towards the urethra, one towards the perineum, and the other towards the left labium. The pains, although not strong, immediately expelled the child. The uterus, after the secundines separated and came away, with no difficulty, contracted and returned, with but little assistance, to its pristine situation. Her recovery was as easy as is the case in any natural labor; she was up and walking about the room in three weeks after her delivery. At the age of fifteen she had a prolapsus uteri, which was reduced after it was washed in a strong decoction of oak bark and dusted with powdered resin.

The second case referred to will be found reported by Dr. Gardner, of this city, in volume twelve, new series, of the American Journal of Sciences for 1846. The patient, a Mrs. Potter, aged 35, was in labor at the full time with her fourth child. After her last confinement she had prolapsus uteri. Within the last four months of her present gestation, the uterus has descended so much, that from three to four inches have been always extruded beyond the vulva. In this condition the parts were found about twelve hours after the commencement of labor. The patient now began to show symptoms of exhaustion from the continuance of pains which did not allow a moment's rest, and various other symptoms began to show themselves indicating convulsions. Delay was considered no longer justifiable; the neck of the uterus was still unobliterated, and the os uteri was dilated to only a moderate degree. The vertex of the child lay in the hollow of the sacrum—the presentation was the third of Baudelocque. The time for active interference having arrived, the short forceps were applied—not however without much trouble, owing to the difficulty of inserting the blades through the narrow orifice of the uterus. In conjunction with a stronger pair, and traction, the head was dislodged and came down, bringing with it the os uteri. Great difficulty was now experienced in withdrawing the forceps. The uterus presented an external mass eight inches in length and five in diameter. The os was two and a half inches in diameter. By means of a bistoury an incision was made two and a half inches in length, soon after which a renewal of pain resulted in the expulsion of a live female child, not, however, without tearing up the incision an inch and a half more. The afterbirth was delivered without difficulty or hæmorrhage; the uterus remained external but contracted down to one-half its previous size. Twelve days after delivery, the uterus having diminished much in size, was returned with but little difficulty. She had a slow and protracted recovery, followed by prolapsus of the uterus.

Such is the history of two cases of a rare and unpleasant complication of labor. Various writers on obstetrics have made mention in their works of this complication. Among others, Smellie, Blundall, and Meigs, may be particularly mentioned.—*N. Y. Journal of Medicine.*

